# Circulating Anti-Sorting Nexins 16 Antibodies as an Emerging Biomarker of Coronary Artery Disease in Patients with Obstructive Sleep Apnea

**DOI:** 10.3390/diagnostics10020071

**Published:** 2020-01-27

**Authors:** Yusuke Katsumata, Jiro Terada, Takuma Matsumura, Ken Koshikawa, Seiichiro Sakao, Go Tomiyoshi, Natsuko Shinmen, Rika Nakamura, Hideyuki Kuroda, Kengo Nagashima, Yoshio Kobayashi, Eiichi Kobayashi, Yasuo Iwadate, Xiao-Meng Zhang, Takaki Hiwasa, Koichiro Tatsumi

**Affiliations:** 1 Department of Respirology, Graduate School of Medicine, Chiba University, Chiba 260-8670, Japan; ahna4462@chiba-u.jp (Y.K.); takumaro_olympus@yahoo.co.jp (T.M.); koshi6488@yahoo.co.jp (K.K.); sakaos@faculty.chiba-u.jp (S.S.); tatsumi@faculty.chiba-u.jp (K.T.); 2 Fujikura Kasei Co.,Ltd., Minato-ku, Tokyo 105-0011, Japan; tomiyoshi@restaff.chiba-u.jp (G.T.); s-natsuko@restaff.chiba-u.jp (N.S.); r-nakamura@fkkasei.co.jp (R.N.); h-kuroda@fkkasei.co.jp (H.K.); 3 Research Center for Medical and Health Data Science, The Institute of Statistical Mathematics, Tokyo 190-8562, Japan; nshi@ism.ac.jp; 4 Department of Cardiovascular Medicine, Graduate School of Medicine, Chiba University, Chiba 260-8670, Japan; yuiryosuke@msn.com; 5 Department of Neurological Surgery, Graduate School of Medicine, Chiba University, Chiba 260-8670, Japan; papez@xa2.so-net.ne.jp (E.K.); iwadatey@faculty.chiba-u.jp (Y.I.); hiwasa_takaki@faculty.chiba-u.jp (T.H.); 6 Department of Biochemistry and Genetics, Graduate School of Medicine, Chiba University, Chiba 260-8670, Japan; grapexiaomeng315@yahoo.co.jp

**Keywords:** atherosclerosis, autoantibody, biomarker, coronary artery, obstructive sleep apnea

## Abstract

Biomarkers are not available for monitoring the onset and progression of coronary artery disease (CAD) in patients with obstructive sleep apnea (OSA), a major risk factor for arteriosclerotic cardiovascular diseases. This study aimed to test for correlation between circulating anti-Sorting Nexins 16 antibody (SNX16-Ab) levels, CAD history and clinical parameters of patients with OSA. Sixty-four healthy donors, 82 adults with OSA, and 96 with acute coronary syndrome (ACS) were studied. Serum samples were collected at diagnostic polysomnography in the OSA group or at the disease onset in the ACS group. Serum SNX16-Ab levels were measured by amplified luminescence proximity homogeneous assay (AlphaLISA), and correlation between SNX16-Ab levels and clinical parameters was analyzed. SNX16-Ab levels and apnea-hypopnea index (AHI) were weakly correlated. Additionally, logistic regression analyses of OSA group identified that elevated SNX16-Ab level associated with the history of CAD. Circulating SNX16-Ab could increase during CAD pathogenesis in patients with OSA. Further prospective studies are required to prove the predictive potential of SNX16-Ab level in CAD onset of patients with OSA.

## 1. Introduction

Obstructive sleep apnea (OSA) is a common sleep-related breathing disorder characterized by recurrent upper airway obstruction, intermittent hypoxia, frequent sleep fragmentation, and intra-thoracic pressure swings during sleep. These nocturnal respiratory disturbances potentially impact the hormone-metabolic, hemodynamic, oxidative, and immuno-inflammatory mechanisms. Endothelial dysfunction and atherosclerosis are likely highly related with the physiological and molecular and features of OSA [1,2,3]. Growing evidence also indicates that OSA associated with potential comorbidities such as obesity, hypertension, lipid disorder, and diabetes mellitus promotes an inflammatory state that increases cardiovascular risk [4,5,6]. Furthermore, the risk of atherosclerosis-related deaths is remarkably increased in patients with severe OSA [6,7,8,9], and untreated severe OSA is an independent risk factor for cardiovascular events [10]. Young. T et al. reported the hazard ratio (95% CI) for cardiovascular mortality with severe OSA patients versus no OSA patients was 5.2 (1.4–9.2) [9]. Continuous positive airway pressure (CPAP), the standard treatment for OSA, improves blood pressure and lipid profile, and reduces cardiovascular mortality [11,12,13]. Marin JM, et al. report that incidence of fatal cardiovascular events in untreated severe OSA (2.13 per 100 person-years) was significantly higher than in OSA treated with CPAP (0.35 per 100 person-years) [10]. However, the CPAP non-adherence rate based on a criterion of ≤4 h of use has been reported at 29% to 83% [14]. Therefore, detection of patients with OSA who need intensive treatment (e.g., increased adherence to CPAP) is essential. To that end, convenient and non-invasive blood biomarkers are required. Although several studies have reported biomarkers of coronary artery disease (CAD) risk in patients with OSA [15,16], there is no consensus on practical biomarkers to date. Recently, a variety of circulating autoantibodies against atherosclerosis-related antigens have been found. Our group has previously reported autoantibodies in the serum of patients with atherosclerosis-related diseases [17,18]. Among them, the circulating autoantibodies against coatomer protein complex subunit epsilon, which can be a biomarker of CAD and stroke risk in patients with OSA [19], and bone morphogenetic protein antagonist neuroblastoma suppressor of tumorigenicity which can be a potential biomarker of cardiovascular risk in patients with OSA were identified [16]. In this study, we identified elevated levels of anti-sorting nexins 16 antibodies (SNX16-Abs; antibodies against a family of cytoplasmic and membrane-associated proteins) after screening the sera of patients with OSA for multiple autoantigens. Subsequently, an amplified luminescence proximity homogeneous assay (AlphaLISA) was used to evaluate the selected antibody levels in the serum of patients with OSA. We tested for correlation between circulating SNX16-Ab levels and the history of CAD and clinical parameters of patients with OSA.

## 2. Materials and Methods

### 2.1. Ethical Approval

The protocol for analyzing serum from participants adhered to the ethical standards and was approved on August 9, 2018 by the Ethical Review Board of the Chiba University, Graduate School of Medicine (approval number 973). This study complied with the 1964 declaration of Helsinki and the subsequent amendments. Before taking blood samples, written informed consent was obtained from all participating patients.

### 2.2. Research Subjects

Eighty-two Japanese adults with a median age of 59 years, 56 men, and 26 women, in whom OSA was diagnosed by PSG at our hospital from June 2012 to January 2014, were recruited. Ninety-six Japanese adults with a median age of 67 years, 81 men and 15 women, in whom ACS was diagnosed at our hospital were recruited at the onset of disease from April 2011 to March 2014. The ACS group includes 72 patients with AMI and 24 with unstable angina pectoris (UAP). Sixty-four HA with a median age of 42.5 years, 38 men and 26 women, and with no history of OSA or CAD were recruited as control group. In our previous study [16], the same research subjects were enrolled to clarify another autoantibody marker, autoantibody against NBL-1, for OSA with the history of CAD. CAD was defined as myocardial infarction or angina pectoris. All HA were recruited at our hospital. In selecting HA, participants with autoimmune diseases, CAD and OSA, according to the absence of OSA symptoms, were excluded.

### 2.3. Clinical Data

Clinical data including age, sex, BMI, hypertension, diabetes, dyslipidemia, smoking status, CAD, and stroke were collected from clinical records. Hypertension, diabetes, and dyslipidemia were defined as the history of those diagnoses or the use of drugs for those diseases. People were divided into three groups based on their smoking history: never smokers, ex-smokers, and current smokers. PSG admission was scored based on the 2007 American Academy of Sleep Medicine alternative criteria [20]. OSA was diagnosed according to the third edition of the International Classification of Sleep Disorders. OSA severity was classified based on AHI values; mild, 5–15; moderate, 15–30, and severe, > 30.

### 2.4. Blood Sample Collection and Experimental Method

Serum samples were collected from OSA group during PSG admission, HA group during medical checkup, and ACS at diagnosis of UAP or AMI during admission for coronary artery bypass graft or percutaneous coronary intervention. The samples were centrifuged at 3000 *g* for 10 min at room temperature and stored at −80°C. A full-length SNX-16 cDNA was expressed using an expression vector pGEX-4T-3 for the glutathione-S-transferase (GST) -tagged SNX-16 protein. The product of the gene was purified as previously described [18,21,22]. AlphaLISA (PerkinElmer, Waltham, MA, USA) was conducted in 384-well microtiter plates (PerkinElmer) containing 2.5 μL of GST or a GST-fusion protein (10 μg/mL) in AlphaLISA buffer (25 mM HEPES, pH 7.4, 0.05% proclin 300, 1 mg/mL dextran 500, 0.1% casein, and 0.5% Triton X-100) and 2.5 μL of 1/100-diluted sera. The mixture was incubated at room temperature for 1 to 14 days. Anti-human IgG conjugated acceptor beads (2.5 μL of 40 μg/mL) and glutathione conjugated donor beads (2.5 μL of 40 μg/mL) were added and the samples were incubated at room temperature in the dark for 14 days. The chemical emission was measured with an EnSpire Alpha microplate reader (PerkinElmer, Waltham, MA, USA) as described previously [21,22]. The target antibody level was measured by subtracting the alpha value of GST control sample from the alpha value of the sample containing GST fusion protein.

### 2.5. Statistical Analysis

All statistical analyses were performed with JMP Pro 12.2.0 software (SAS Institute Inc., Cary, NC, USA). The significance of differences in baseline characteristics between groups was analyzed using the Kruskal–Wallis test for categorical data and Mann–Whitney U or Kruskal–Wallis test for numerical data. The significance of differences among HA, mild OSA, moderate OSA, and severe OSA group was analyzed using the Steel–Dwass test as a post hoc analysis. Subgroup analyses were performed for SNX16-Ab levels by OSA severity or the history of CAD. Correlation of SNX16-Ab level and clinical data of OSA group was evaluated using Spearman correlation analysis. The SNX16-Ab level cut-off value for the history of CAD in OSA group was calculated by ROC curve analysis to maximize the sum of specificity and sensitivity. Multivariate and univariate logistic regression analysis was used to identify variables that could predict patients with the history of CAD. Statistical significance was defined as a *p* value < 0.05, and all tests were two-sided.

### 2.6. Ethics Approval and Consent to Participate

All experiments were performed with the approval of the Animal Experiment Ethics Committee of the Murayama Medical Center and in accordance with the Guiding Principles for the Care and Use of Animals of the Physiological Society of Japan.

### 2.7. Availability of Data and Material

The datasets generated during the current study are available from the corresponding author on reasonable request.

## 3. Results

### 3.1. Clinical Characteristics

Characteristics of the healthy adults (HA), OSA, and acute coronary syndrome (ACS) group are shown in Table 1. The OSA and ACS group were significantly older than the HA group. ACS group included more male patients than HA group. The history of CAD, diabetes, dyslipidemia, and hypertension was more frequently observed in the ACS and OSA group than in the HA group.

### 3.2. Difference in SNX16-Ab Level for Each Group

**Figure 1:**
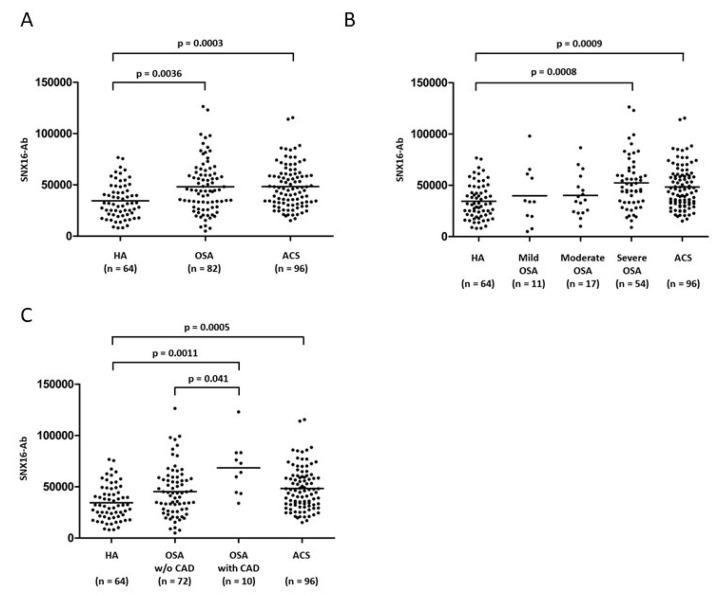
Difference in SNX16-Ab level for each group. The level of SNX16-Ab, measured by AlphaLISA, was compared among the three groups; OSA, ACS, and HA group. (**A**) Kruskal–Wallis test revealed significant differences among the three groups (*p* < 0.001). Steel–Dwass test revealed significant differences between patients with ACS or OSA versus HA group. (**B**) Significant differences were observed between patients with severe OSA group and HA group and (**C**) between patients with OSA with CAD versus HA group. Horizontal lines represent medians. After performing the Kruskal–Wallis test, the Steel–Dwass test was performed on all pairs; *p* values of Steel–Dwass test were described. Since the number of groups to be analyzed was different, the *p* values were different even between the same groups. ACS: acute coronary syndrome; CAD: coronary artery disease; HA: healthy adults; OSA: obstructive sleep apnea; SNX16-Abs: antibodies against SNX16; w/o: without.

After screening the serum of patients with OSA for multiple candidates of autoantigens recognized by IgG antibodies using protein arrays, we selected and identified SNX16-Abs that were elevated. As shown in Figure 1A, the serum levels of SNX16-Ab in the OSA and ACS group were significantly higher than those in HA group. SNX16-Ab levels between the OSA group and ACS group were not significantly different. (*p* = 0.9314). SNX16-Ab levels in patients with severe OSA or the history of CAD were significantly higher than those in HA group (*p* < 0.001, Figure 1B,C). The SNX16-Ab levels in OSA group with no history of CAD or with mild-to-moderate severity were similar to those in HA group.

### 3.3. Correlation of SNX16-Ab Level and Clinical Data of OSA Group

**Figure 2:**
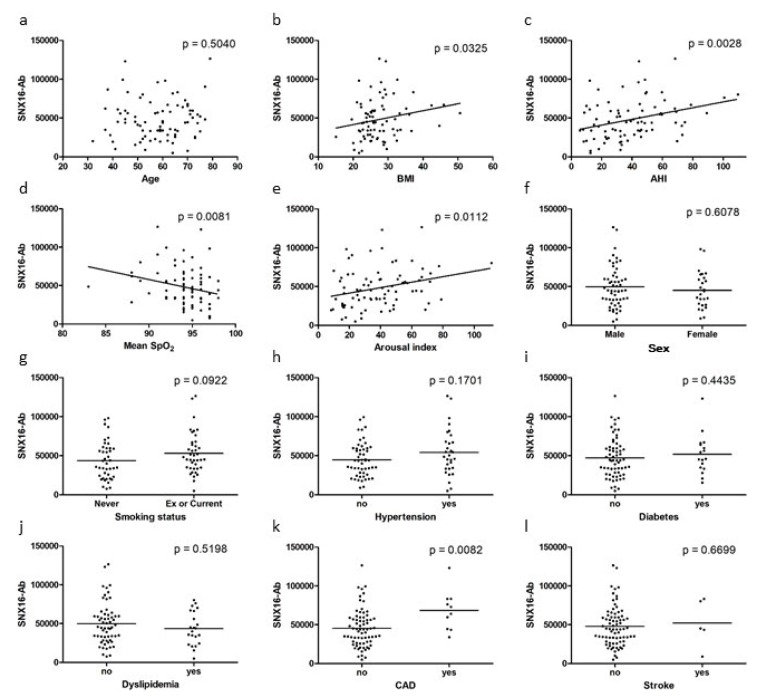
Association between serum SNX16-Ab level and clinical data in the OSA group. (**a**) Association between SNX16-Ab level and age, (**b**) body mass index, (**c**) apnea-hypopnea index, (**d**) mean SpO_2_, (**e**) arousal index, (**f**) sex, (**g**) smoking status, (**h**) hypertension, (**i**) diabetes, (**j**) dyslipidemia, (**k**) coronary artery disease, and (**l**) stroke were analyzed. Spearman’s correlation analysis (**a**–**e**), Mann–Whitney U test (**f**–**l**) were used. Horizontal lines represent medians. AHI: apnea-hypopnea index; BMI: body mass index; CAD: coronary artery disease; SNX16-Ab: antibodies against SNX16; SpO_2_: oxygen saturation of peripheral artery.

The association between SNX16-Ab levels and the clinical data of the OSA group and ACS group are shown in Figure 2 and Supplementary Figure S1. Weak but significant correlation was observed between SNX16-Ab level and apnea-hypopnea index (ρ = 0.32, *p* = 0.003, Figure 2c). A very weak significant association was observed between SNX16-Ab level and body mass index (BMI) (ρ = 0.24, *p* = 0.003, Figure 2b), mean SpO_2_ (ρ = −0.29, *p* = 0.008, Figure 2d) and arousal index (ρ = 0.28, *p* = 0.011, Figure 2e). A significantly elevated SNX16-Ab levels were found in patients with CAD than in those without CAD (*p* = 0.008, Figure 2k). No significant association was observed between SNX16-Ab levels and age, sex, smoking status, history of diabetes, hypertension, dyslipidemia, and stroke. However, univariate analysis of association between the SNX16-Ab level and each factor in ACS group suggests smoking status as an influencing factor (Supplementary Figure S1). Multiple regression analysis suggests correlation between smoking status and SNX16-Ab level in ACS group (*p* = 0.036), not in OSA group (*p* = 0.19).

**Figure 3:**
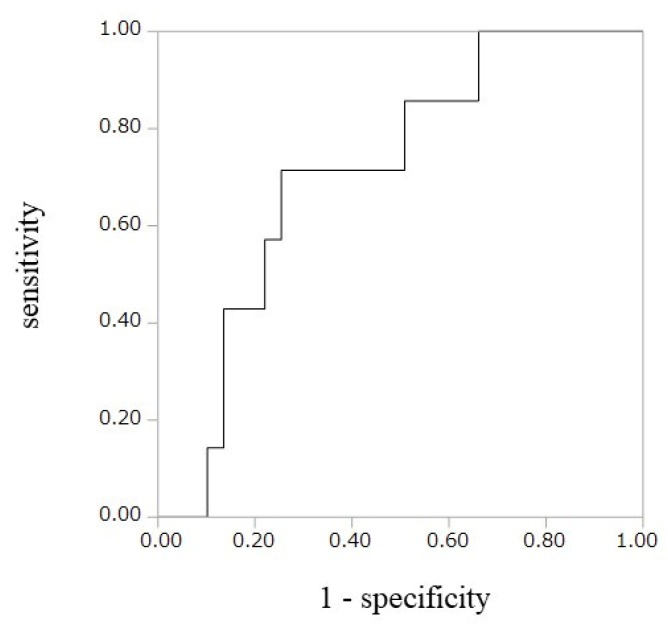
ROC curve demonstrated predictive value of SNX16-Ab for the history of CAD in OSA group. Cutoff value of 59,735 showed a sensitivity of 71.4% and specificity of 74.6% (*n* = 82; no. of events = 10) for the history of CAD. The area under the curve was 0.712.

We used univariate and multivariate logistic regression models to clarify the strength of the correlation between the history of CAD and clinical parameters in the OSA group, as shown in Table 2. An optimal cutoff value of SNX16-Ab for predicting the presence of the history of CAD in the OSA group was 59,735 by receiver operating characteristic (ROC) curve analysis, with a sensitivity of 71.4%, a specificity of 74.6%, area under the curve of 0.712 (Figure 3). The results of applying the SNX16-Ab cut-off value in each group are as shown in Supplementary Table S1. The univariate logistic regression clarified a correlation between the history of CAD and elevated SNX16-Ab level using the SNX16-Ab cut-off value [odds ratio (OR): 8.87, 95% confidence interval (CI): 2.19–45.1, *p* = 0.002]. The multivariate logistic regression analysis using parameters with *p* < 0.10 by univariate analysis (elevated SNX16-Ab level and severe OSA) revealed significant correlation between elevated SNX16-Ab level and the history of CAD (OR: 8.61, 95% CI: 2.07–45.0, *p* = 0.003).

## 4. Discussion

This study presents two major findings of circulating SNX16-Ab in patients with OSA. First, serum levels of SNX16-Ab are significantly higher in OSA and ACS group compared to those in HA (control) group. Second, subgroup analyses show especially elevated serum levels of SNX16-Ab in severe OSA group and OSA with the history of CAD group. Thus, our results suggest that serum SNX16-Ab may increase in connection with CAD pathogenesis in patients with preexisting OSA.

The sorting nexins (SNXs), a family of cytoplasmic and membrane-associated proteins, are responsible for sorting membrane proteins [23]. Even though a clear evidence between SNXs-CAD pathogenesis-OSA is lacking, potential mechanisms might be considered. For instance, epidermal growth factor receptor (EGFR), one of other sorting membrane proteins, is expressed in endothelial cells, vascular smooth muscle cells, cardiomyocytes and macrophages [24], and causes downstream activation of transcription factors such as nuclear factor-κB. It also stimulates pro-inflammatory gene transcription in macrophages, playing a role in foam cell transformation, cellular dysfunction, and proliferation of vascular smooth muscle cells [25]. On the other hand, SNX16 is localized in the early and recycling endosomes via its Phox domain, and it may negatively regulate EGFR-mediated signaling by potentiating the degradation of EGFR [26]. In addition, SNX16 overexpression in late endosomes is reported to cause cholesterol accumulation. Generally, low-density lipoproteins (LDL)-derived cholesterol taken up via the LDL receptor is transported to the cell membrane, endoplasmic reticulum, and mitochondria by endocytosis. However, SNX16 overexpression in late endosomes inhibit cholesterol transport, accumulate cholesterol, and may cause arteriosclerosis. [27] Preceding cardiovascular disease or stroke, atheromatous plaques are partially ruptured, leaking accumulated SNX16 into the blood. Subsequently, autoantibody against SNX16 may be produced by such repetitive SNX16 leakage. In this study, elevated SNX-16 Ab correlated with the history of CAD and OSA parameters such as frequent arousal, intermittent hypoxia (i.e., AHI), and obesity (i.e., BMI). However, since these results and the current literature do not reveal a direct molecular or clinical link between SNX16 and arteriosclerosis in patients with OSA, this question remains to be explored.

In regard to autoantibody production, it generally takes several weeks to produce IgG antibodies after antigen exposure. In this study, the sera were collected from the ACS group at the onset of acute myocardial infarction (AMI) diagnosis. Therefore, SNX16-Ab might have been produced several weeks before the onset of AMI. This suggests that SNX16-Ab level could be useful in predicting the onset of AMI.

Recently, several potential biomarkers for evaluating arteriosclerosis have been reported. Oxidized low-density lipoproteins (oxLDL) are reported to be related to the progression of arteriosclerosis [28]. Circulating anti-oxLDL antibodies and oxLDL may be optimal biomarkers for cardiovascular disease risk, however, their true significance remains uncertain [29]. Although increased levels of autoantibody against heat shock protein (Hsp) family are reported in patients with arteriosclerotic diseases [30,31], their ability to predict the occurrence of arteriosclerotic diseases remains unclear. Sun et al. [32] reported that higher circulating ESM-1, previously named endothelial cell-specific molecule-1, correlated with the presence of CAD in patients with OSA. Furthermore, several studies demonstrated that the level of placental growth factor (PIGF), as a biomarker of ACS, was significantly higher in patients with OSA [33]. Thus, although a single marker might not be sufficient as a predictive biomarker of ACS in patients with OSA, a combination of several biomarkers including SNX-16 Ab may have a predictive potential in future clinical practice.

This study includes several limitations. First, the existence of OSA in patients with ACS or in HA group was excluded only by medical history and not by diagnostic testing such as polysomnography (PSG). Subclinical OSA was not completely excluded in the ACS or HA group. Additionally, although the analysis in the OSA group has denied the correlation between age, sex, smoking status, hypertension, diabetes, dyslipidemia and SNX16-Ab level (see Figure 2), potential confounding factors in between the OSA, ACS and HA group were not completely excluded. Second, the SNX16-Ab cutoff for the history of CAD in the OSA group might not be accurate, since the number of populations with OSA and CAD was small. Third, sex was not included as a covariate in the logistic regression analysis, because all patients with CAD in the OSA group were male. Forth, the severity of atherosclerosis was not evaluated by conventional physiological tests. Fifth, potential confounding factors between the patients and HAs were not adjusted. Finally, only Japanese patients participated in this study. Further studies are needed in other ethnic groups.

In summary, our subjects with OSA had higher SNX16-Ab levels than healthy subjects. SNX16-Ab levels were significantly elevated in the OSA group with the history of CAD or severe OSA compared to HA group. Based on our results, we suggest that SNX16-Ab could increase in connection with the pathogenesis of CAD in patients with OSA. However, additional prospective studies are warranted to prove the predictive potential of SNX16-Ab level for the onset of CAD in the OSA group. The results of this study might evoke further studies to research biomarkers involved in the devastating progression of cardiovascular diseases in OSA.

## Figures and Tables

**Table 1:** Patient characteristics.

	HA (*n* = 64)	OSA (*n* = 82)	ACS (*n* = 96)
Age	42.5 (35.3–55.8)	59.0 ^***^ (49.8–66.5)	67.0 ^***^ (60.0–73.0)
Male (%)	59.4	68.3	84.4 ^***^
BMI (kg/m^2^)	23.1 (20.6–25.5)	25.9 ^***^ (23.9–29.4)	23.4 (21.3–25.2)
OSA severity (%)			
mild		13.4	
moderate		20.7	
severe		65.9	
AHI (/h)		36.7 (22.6–50.4)	
Mean SpO_2_ (%)		94 (93.0–96.0)	
Mean SpO_2_ (%)		78 (69.0–83.0)	
Arousal Index (/h)		37.3 (22.2–50.3)	
Smoking status (%)			
Never	70	51.2	31.3
Ex	16.7	41.5	39.6
Current	13.3	7.3 ^**^	29.2 ^***^
Hypertension (%)	12.5	36.6 ^**^	46.9 ^***^
Diabetes mellitus (%)	1.6	20.7 ^**^	17.7 ^**^
Dyslipidemia (%)	3.1	26.8 ^*^	14.6 ^*^
CAD (%)	0	12.2 ^**^	14.6 ^***^
Stroke (%)	0	6.1	5.2

Data are medians (interquartile range) for numerical data and *n* (%) for categorical data. * *p* < 0.05 versus HA, ** *p* < 0.01 versus HA, *** *p* < 0.001 versus HA. ACS: acute coronary syndrome; AHI: apnea hypopnea index; BMI: body mass index; CAD: coronary artery disease; HA: healthy adults; OSA: obstructive sleep apnea; SpO_2_: oxygen saturation of peripheral artery.

**Table 2:** Logistic regression of correlation between the history of CAD and clinical parameters in OSA group.

	Univariate Analysis			Multivariate Analysis		
	OR	95% CI	*p* value	OR	95% CI	*p* value
Age (per year)	1.04	0.97–1.11	0.26			
BMI (≥25)	0.80	0.21–3.36	0.75			
Smoking	2.76	0.71–13.6	0.15			
Hypertension	0.71	0.14–2.81	0.64			
Diabetes	3.02	0.69–12.2	0.13			
Dyslipidemia	1.20	0.24–4.79	0.81			
Severe SAS	5.4	0.94–102.3	0.06	5.12	0.81–100.8	0.088
SNX16-Ab (≥ 59735)†	8.87	2.19–45.1	0.0021	8.61	2.07–45.0	0.0029

†SNX16-Ab cutoff was 59735 evaluated by ROC curve analysis. AHI: apnea-hypopnea index; BMI: body mass index; CAD: coronary artery disease; CI: confidence interval; OR: odds ratio; OSA: obstructive sleep apnea; SAS: sleep apnea syndrome; SNX16-Ab: antibodies against SNX16.

## Supplementary Materials

The following are available online at https://www.mdpi.com/2075-4418/10/2/71/s1, Figure S1: Association between serum SNX16-Ab level and clinical data in ACS group. (**a**) Association between SNX16-Ab level and smoking status, (**b**) Hypertension, (**c**) Diabetes, and (**d**) Dyslipidemia were analyzed. Mann-Whitney U test (a-d) were used. Horizontal lines represent medians. SNX16-Ab: antibodies against SNX16. Table S1: SNX16-Ab level of each group.

## Abbreviations

ACS	Acute Coronary Syndrome
AHI	Apnea-Hypopnea Index
AlphaLISA	Amplified Luminescence proximity homogenous Assay
AMI	Acute Myocardial Infarction
BMI	Body Mass Index
CAD	Coronary Artery Disease
CPAP	Continuous Positive Airway Pressure
EGFR	Epidermal Growth Factor Receptor
GST	Glutathione S-transferase
HA	Healthy Adults
LDL	Low-density lipoproteins
OSA	Obstructive Sleep Apnea
oxLDL	Oxidized low-density lipoproteins
PSG	Polysomnography
PIGF	placental growth factor
ROC	Receiver Operating Characteristic
SNXs	Sorting nexins
SNX16-Ab	Antibody against SNX16
UAP	Unstable Angina Pectoris
