# Supplementary material for: Circulating Anti-Sorting Nexins 16 Antibodies as an Emerging Biomarker of Coronary Artery Disease in Patients with Obstructive Sleep Apnea
Source: Diagnostics (Basel). 2020 Jan 27;10(2):71. doi: 10.3390/diagnostics10020071 (PMC7168932; doi:10.3390/diagnostics10020071)
Supplement: Supplementary file 1 [file diagnostics-10-00071-s001.zip › diagnostics-692132-SI/Supplementary Table S1.docx]

Supplementary Table S1. SNX16-Ab level of each group.

|  | SNX16-Ab level ≥ 59735 (%) | SNX16-Ab level < 59735 (%) | SNX16-Ab level |
| --- | --- | --- | --- |
| HA group | 10.9 % | 89.1 % | 35433 (21051 – 48396)  (60.0-73.0) |
| OSA group | 26.8 % | 73.2 % | 48169 (29573 – 60741) |
| ACS group | 26.0 % | 74.0 % | 48347 (32631 – 60131) |

Data are medians (interquartile range) for numerical data and *n* (%) for categorical data. ACS: acute coronary syndrome; HA; healthy adults OSA: obstructive sleep apnea; SNX16-Ab: antibodies against SNX16.
