# Supplementary figures and images for: Circulating Anti-Sorting Nexins 16 Antibodies as an Emerging Biomarker of Coronary Artery Disease in Patients with Obstructive Sleep Apnea
Source: Diagnostics (Basel). 2020 Jan 27;10(2):71. doi: 10.3390/diagnostics10020071 (PMC7168932; doi:10.3390/diagnostics10020071)

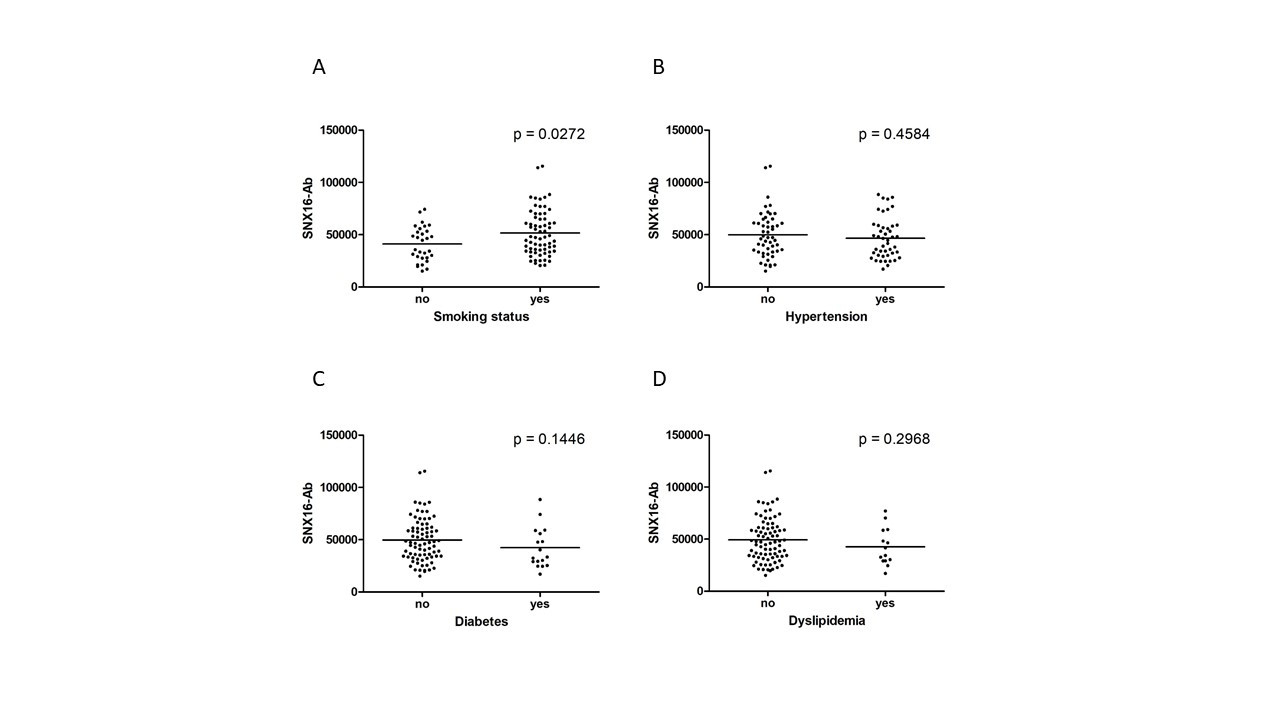

Supplement: Supplementary file 1 [file diagnostics-10-00071-s001.zip › diagnostics-692132-SI/Supplementary_Figure_S1.jpg]
